# Biomedical named entity recognition based on multi-cross attention feature fusion

**DOI:** 10.1371/journal.pone.0304329

**Published:** 2024-05-28

**Authors:** Dequan Zheng, Rong Han, Feng Yu, Yannan Li

**Affiliations:** 1 Harbin University of Commerce, Harbin, China; 2 Heilongjiang Provincial Key Laboratory of Electronic Commerce and Information Processing, Harbin, China; Shanghai Maritime University, CHINA

## Abstract

Currently, in the field of biomedical named entity recognition, CharCNN (Character-level Convolutional Neural Networks) or CharRNN (Character-level Recurrent Neural Network) is typically used independently to extract character features. However, this approach does not consider the complementary capabilities between them and only concatenates word features, ignoring the feature information during the process of word integration. Based on this, this paper proposes a method of multi-cross attention feature fusion. First, DistilBioBERT and CharCNN and CharLSTM are used to perform cross-attention word-char (word features and character features) fusion separately. Then, the two feature vectors obtained from cross-attention fusion are fused again through cross-attention to obtain the final feature vector. Subsequently, a BiLSTM is introduced with a multi-head attention mechanism to enhance the model’s ability to focus on key information features and further improve model performance. Finally, the output layer is used to output the final result. Experimental results show that the proposed model achieves the best F1 values of 90.76%, 89.79%, 94.98%, 80.27% and 88.84% on NCBI-Disease, BC5CDR-Disease, BC5CDR-Chem, JNLPBA and BC2GM biomedical datasets respectively. This indicates that our model can capture richer semantic features and improve the ability to recognize entities.

## 1. Introduction

**Fig 1:**
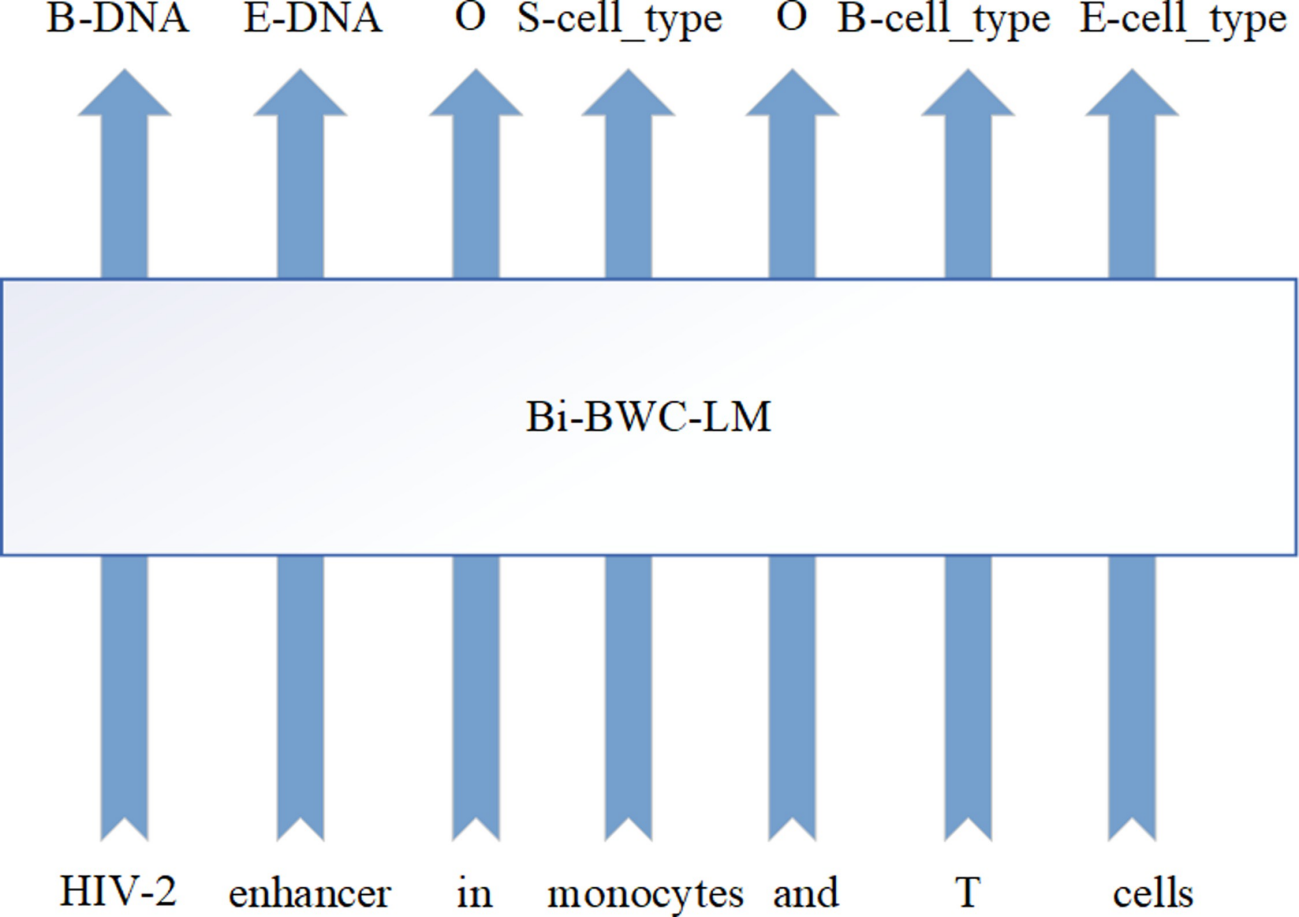
An example of BioNER.

Named entity recognition [1, 2] refers to the identification of specific entities in text, such as people, places, and organizations, with the aim of locating entity mentions from unstructured text and classifying them into predefined categories. However, unlike in general domains, the field of biomedicine, as an interdisciplinary subject that combines methods and theories from multiple disciplines such as medicine and biology, contains a vast amount of scientific literature that records key information such as clinical studies, drug treatments, and gene expressions. This information is written for domain experts and typically requires a broader domain-specific knowledge for information extraction. Additionally, due to the vast amount of biomedical literature, manual processing of this text becomes a laborious and time-consuming task. Despite the fact that numerous manual annotation efforts have been organized internationally to extract biomedical concepts and information from text, and store the extracted information in structured knowledge resources such as Swiss-Prot [3] and GenBank [4], the rapid growth of literature data and the emergence of infectious diseases such as SARS-CoV-2 and monkeypox have made it increasingly important to develop automated and high-performance NER (Named Entity Recognition) methods to assist in retrieving, organizing, and managing large amounts of biomedical data and information. BioNER (Biomedical Named Entity Recognition) is a method that uses natural language processing techniques to annotate entities such as diseases, genes, proteins, etc. In text, and is also a crucial subtask for subsequent tasks such as biological literature retrieval [5] and biological question-answering systems [6]. As shown in Fig 1, a sample of BioNER on a biomedical text is given.

This article proposes a BioNER method based on multi-cross attention feature fusion, named Bi-BWC-LM. Firstly, we perform three rounds of cross-attention feature fusion on character and word features. Subsequently, a BiLSTM is introduced with a multi-head attention mechanism to enhance the model’s ability to focus on key information features and further improve model performance. Finally, the output layer is used to output the final result.

In this article, Section 1 introduces the introduction of the paper, followed by Section 2 which presents the background of the paper, mainly including related work and models pertinent to this study. Subsequently, Section 3 focuses on the model structure proposed in this paper, while Section 4 details the key experimental results. Finally, Section 5 summarizes the conclusions of this paper, discusses its limitations, and provides an outlook for future work.

The main contributions of this article are as follows:

1. A three-cross attention feature fusion method is proposed, which involves cross-attention feature fusion between DistilBioBERT and CharCNN, as well as between DistilBioBERT and CharLSTM. The fused features are then subject to another round of cross-attention feature fusion.
2. Multi-head attention is added after the BiLSTM to address the challenges of capturing long-distance dependencies in BiLSTM and enhance the representation ability of input information. It selects more critical information from numerous textual data from a multi-level perspective.
3. The model is trained using a combined loss function, which incorporates both cross-entropy loss and Focal Loss. This approach considers the issue of data imbalance, increases the penalty for misclassification, and provides an adaptive adjustment mechanism.
4. Experimental results and ablation studies conducted on five biomedical datasets, including NCBI-Disease, BC5CDR-Disease, BC5CDR-Chem, BC2GM, and JNLPBA, demonstrate that the proposed model is highly effective in improving the recognition performance of biomedical named entities.

## 2. Background

In this section, we first introduce the relevant work of this article, and then introduce the relevant models used in the tasks of this article.

### 2.1. Related work

Traditional biomedical named entity recognition methods can be divided into rule-based and dictionary-based methods. However, rule-based [7] and dictionary-based [8] methods often have strong dependence on domain knowledge, poor scalability and portability, and often require a significant amount of time to develop rules and establish dictionaries. With the increase in data volume, more and more researchers are trying to use machine learning methods to handle BioNER tasks, such as Hidden Markov Model [9], Support Vector Machine model [10], Maximum Entropy model [11], and Conditional Random Fields model [12]. However, traditional machine learning methods typically require large amounts of labeled data for training, have high data dependence, require extensive feature engineering, and have difficulty with context modeling and handling unknown entities. At this point, deep learning methods provide a simpler solution to solve these problems.

CharCNN [13] and CharLSTM [14] are two character-level neural network models that can infer representations of unseen words and share information at the morpheme level. CharCNN uses convolutional and pooling operations to extract features from the input character sequence, with a focus on extracting local features, while CharLSTM models the entire character sequence using LSTM units, enabling the learning of more global and long-range context information. Ma et al [15] proposed a BiLSTM-CNN-CRF model that first uses CNN to extract character-level features, then uses a BiLSTM-CRF model to further extract contextual features and output the results. Mohsen Asghari et al [16] used four different embedding methods, RNN-Character level, CNN-Character level, LookUp, and Self-attended encoder to study the recognition of biomedical text. Kuru et al [17] proposed CharNER, a character-level tokenizer for named entity recognition that is language-independent. It used LSTM to extract character-level representations and outputs the label distribution for each character rather than each word. Then, the word-level labels are obtained from the character-level labels. M. Cho et al. [18] combined CNN and BiLSTM as feature information extraction, and then added an attention mechanism to the BiLSTM-CRF model for further extraction and recognition of BioNER.

To enhance the feature extraction ability of deep learning models, Vaswani et al [19] proposed the Transformer mechanism. The Transformer mechanism abandoned the structure of recurrent neural networks and directly modeled sequences using the Attention mechanism, greatly accelerating the parallel computing capability of the model. The attention mechanism first achieved success in the field of machine translation [20], and later began to be widely used in various aspects of natural language processing due to its excellent performance in neural network structures. Attention mechanism models can measure the importance of different information features based on information weights, strengthen key information, weaken useless information, and identify key features that affect the final model performance by analyzing the global context. As the name suggests, it allocate weights to text data inputs based on the level of attention paid to words. Bahdanau et al [21] first applied the Attention mechanism to the NER task and achieved excellent results. Subsequently, more and more scholars began to use this method to deal with NER tasks.

The introduction of BERT [22] has greatly advanced the field of natural language processing and achieved excellent performance on multiple tasks. However, with the emergence of various specialized domains, BioBERT [23], a domain-specific language representation model specifically trained on biological medical corpus, has been developed in the field of biomedicine. However, BioBERT has a large number of parameters and requires a large amount of resources to use them in practical environments, which often limits its applicability. Therefore, DistilBioBERT [24] was proposed through knowledge distillation. This model uses a 6-layer transformer and has approximately 65 million parameters. Compared with large models, this model has significantly faster training speed and far fewer parameters, and its performance is almost indistinguishable across different biological medical tasks.

### 2.2. Related models

#### 2.2.1. Convolutional neural networks

CNN (Convolutional Neural Networks) originally designed for analyzing visual images, possess the core feature of convolutional operations, enabling sliding window calculations on images. Through convolutional kernels and pooling layers, CNN extracts features from the images. Subsequently, CNN has been widely applied to natural language processing tasks such as text classification, named entity recognition, and sentiment analysis. CNN captures local dependencies between words and crucial lexical information in sentences through convolutional and pooling operations.

CharCNN, short for Character-level Convolutional Neural Network, is a model specifically designed for processing text data at the character level. Instead of taking words or phrases as input, CharCNN models text data from a character perspective. Through convolutional and pooling operations, it extracts meaningful local and global features, and projects these features onto corresponding labels or categories using fully connected layers. Therefore, CharCNN exhibits superior robustness and generalization capabilities when dealing with text data.

#### 2.2.2. Bidirectional long short-term memory

RNN (Recurrent Neural Networks) are a type of neural network designed to better process sequential information, especially suitable for handling text, speech, and other sequential data. LSTM (Long Short-Term Memory) is an extension of RNN, which adds forget gates, input gates, output gates, and internal memory units to address the gradient vanishing problem inherent in RNN. Subsequently, BiLSTM (Bidirectional Long Short-Term Memory) emerged and gradually became a mainstream model. In BiLSTM, each feature vector passes through a two-layer LSTM, capturing past and future information from different directions. The two independent hidden states are then concatenated to form the final contextual representation. Unlike traditional LSTM, which only considers forward information from the input sequence and can only access information prior to a given time point, BiLSTM takes into account both forward and backward information from the input sequence. This allows BiLSTM to access contextual information from the entire sequence at any time point. The bidirectional nature of BiLSTM also enables it to capture richer contextual information, thereby better understanding the overall structure and semantics of the input sequence. This makes BiLSTM perform better in tasks that require contextual understanding.

CharLSTM, is a text generation model based on LSTM that operates at the character level. Compared to traditional word-level text generation models, CharLSTM is better able to handle semantically ambiguous phrases and complex grammatical structures, resulting in more accurate and fluent text generation. CharLSTM applies LSTM to character-level text generation tasks, breaking down the input text into characters, and feeding each character as a time step into the LSTM.

#### 2.2.3. DistilBioBERT

DistilBioBERT is a pre-trained language model specifically designed for biomedical text processing tasks. It learns knowledge from BioBERT through knowledge distillation. The loss function of DistilBioBERT consists of three weighted losses. The first loss is the standard cross-entropy loss for the MLM (Masked Language Model) objective. This loss helps the model learn contextual representations of vocabulary. By minimizing this loss, the model is able to capture rich lexical semantics. As shown in Eq (1). The second loss is the KL divergence loss of the teacher model’s output, which is used here to measure the difference between the probability distribution of the student model’s output and the probability distribution of the teacher model’s output. As shown in Eq (2). The third loss function is an optional one. It aligns the final hidden states of the teacher and student models through cosine embedding loss, ensuring that the internal representation of the student model closely resembles that of the teacher model. This ensures that the student model can capture similar semantic and contextual information as the teacher model. As shown in Eq (3). The overall loss formula is presented in Eq (4).


$$L_{mlm}(X,Y)=-\sum_{n=1}^{N}W_{n}\left(\sum_{i=1}^{\left|V\right|}Y_{i}^{n}ln\left(f_{s}{\left(X\right)}_{i}^{n}\right)\right)$$ (1)

Among them, X represents the input to the model, and Y denotes the labels for the MLM task. Y is a set of N one-hot vectors, each with a size of |V|, where |V| is the vocabulary size of the model, and N is the number of input tokens. For masked tokens, the value of *W*_*n*_ is 1, and for other tokens, its value is 0. *f*_*s*_ represents the student model.


$$L_{SoftMLM\;}(X)=-\sum_{n=1}^{N}W_{n}D_{KL}\left(f_{t}{\left(X\right)}_{i}^{n}∥f_{s}{\left(X\right)}_{i}^{n}\right)$$ (2)

Among them, *f*_*t*_ represents the teacher model.


$$L_{align\;}(X)=\frac{1}{N}\sum_{n=1}^{N}1-\emptyset \left(h_{t}{\left(X\right)}^{n},\;h_{s}{\left(X\right)}^{n}\right)$$ (3)

Among them, *h*_*t*_ and *h*_*s*_ represent the functions that output the last hidden states of the teacher and student models, respectively, while ∅ denotes the cosine similarity function.


$$L(X,Y)=\alpha_{1}L_{mlm}(X,Y)+\alpha_{2}L_{softMLM}(X)+\alpha_{3}L_{ALIGN\;}(X)$$ (4)

Among them, *α*_1_, *α*_2_, and *α*_3_ represent the weighting terms for the different combinations of losses, respectively. In this chapter, the settings are *α*_1_ = 2.0, *α*_2_ = 5.0, and *α*_3_ = 1.0.

#### 2.2.4. Attention mechanism

In deep learning, the Attention mechanism is an important technique, especially achieving remarkable results in natural language processing tasks. The Attention mechanism can assist neural networks in automatically learning, weighting, and focusing on key information when processing input sequences.The Self-Attention mechanism is a variant of the Attention mechanism that does not rely on external information to calculate attention weights. Instead, it uses information internal to the input sequence for computation, enabling the model to better capture long-distance dependencies when processing sequential data.

The Self-Attention mechanism can sometimes over-focus on its own position when encoding the current location, leading to the proposal of the Multi-Head Attention mechanism to address this issue. Multi-Head Attention is formed by combining multiple Self-Attention components. In the Multi-Head Attention mechanism, h sets of q (query), k (key), and v (value) are used to obtain h different feature representations. The outputs of these h parallel Self-Attention components are then concatenated, and finally, the concatenation is passed through a fully connected layer for dimensionality reduction. The specific expressions for Multi-Head Attention are given in Eqs (5) and (6).


$$MultiHead\;(Q,K,V)=Concat\;\left(head_{1},\;head_{2},\ldots ,\;head_{h-1},\;head_{h}\right)W^{O}$$ (5)

$$head_{i}=Attention\left(QW_{i}^{Q},\;KW_{i}^{K},\;VW_{i}^{V}\right)$$ (6)

Among them, h represents the number of heads, and $W_{i}^{Q},W_{i}^{K},W_{i}^{V},W^{o}$ are learnable linear transformation parameters.

Cross-Attention is important extension of the Self-Attention mechanism. The key difference between Cross-Attention and Self-Attention lies in the input: Self-Attention operates on a single embedded sequence, where Q (Query), K (Key), and V (Value) come from the same input sequence. On the other hand, Cross-Attention correlates information from two different input sequences. In this case, one sequence serves as the Q input, while the other sequence provides the K and V. Alternatively, one sequence can be used for both Q and V, while the other sequence provides the K. Assuming we have two input sequences *S*_1_ and *S*_2_, the specific calculation process is outlined in Eqs (7–10).


$$Q=S_{1}W^{Q}$$ (7)

$$K=S_{2}W^{K}$$ (8)

$$V=S_{2}W^{V}$$ (9)

$$CrossATT(Q,K,V)=softmax\left(\frac{S_{1}W^{Q}{\left(S_{2}W^{K}\right)}^{T}}{\sqrt{d_{S_{2}W^{K}}}}\right)S_{2}W^{V}$$ (10)

Among them, *W*^*Q*^, *W*^*K*^, and *W*^*V*^ represent three trainable parameter matrices, and $d_{S_{2}W^{K}}$ denotes the dimensionality of *S*_2_*W*^*K*^.

## 3. Model construction

This section describes the architecture of the proposed Bi-BWC-LM model. First, we introduce the overall architecture of the model, and then provide a detailed description of each layer of the model.

### 3.1. General architecture of the model

**Fig 2:**
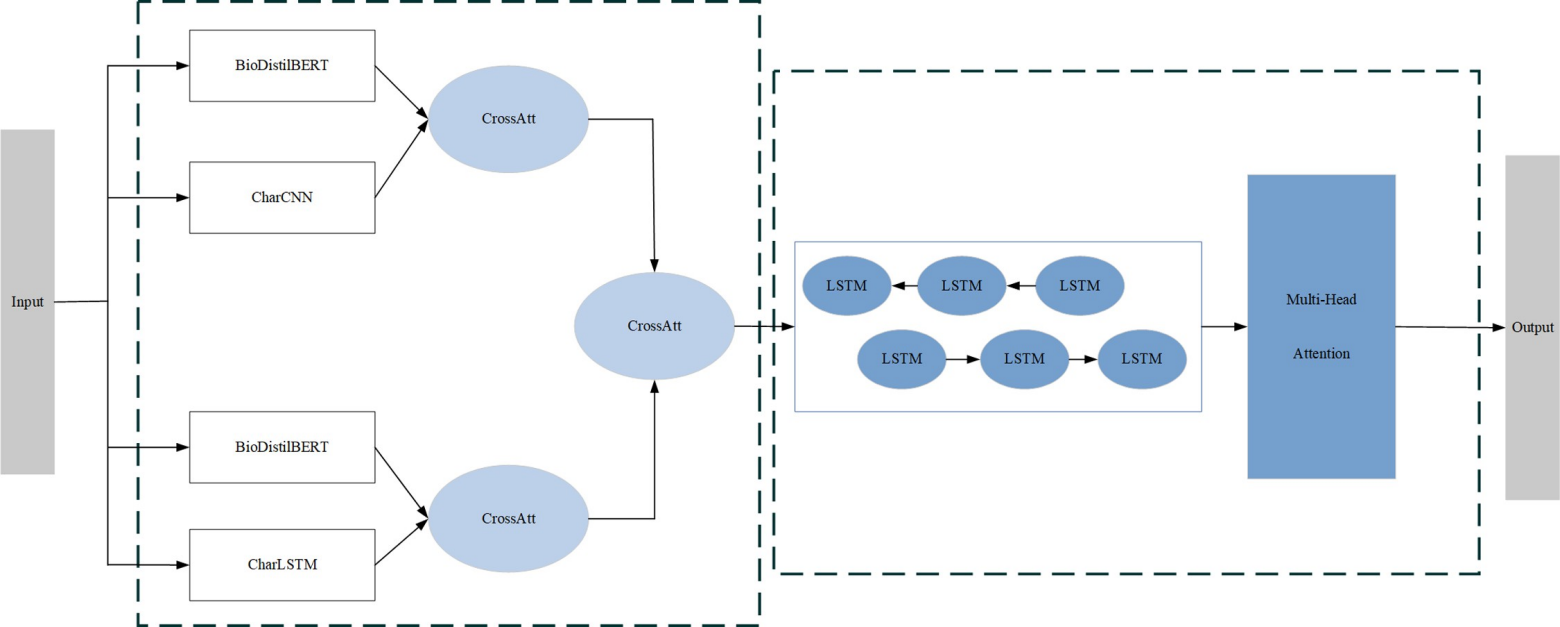
Overall structure of the model.

As shown in Fig 2, the model receives a sentence at the input layer, where all words in the sentence are standardized to lowercase. After the input layer, we use a dual encoder to integrate word embeddings from DistilBioBERT, a pre-trained model that generates dynamic word vectors with contextual semantic information, with character embeddings from CharCNN and CharLSTM. This cross-attention fusion between the word and character embeddings compensates for the shortcomings of CharCNN alone and further improves the word embedding expression, while also avoiding the Out-Of-Vocabulary problem. Then, we perform another cross-attention fusion on the two features after the cross-attention fusion, generating a word vector that is input into the context information layer. The context information layer uses a bidirectional LSTM to capture available features. Subsequently, we use multi-head attention to enable the model to pay attention to important token information in the sentence. This allows the model to better consider the relationships between words and interactions between different words when learning sentence representations, which improves the boundary recognition and classification accuracy of named entities. Finally, the model outputs the final result through an output layer, and is trained using a joint loss function.

### 3.2. Feature extraction layer

All the words in the sentence are processed through the pre-trained model DistilBioBERT to obtain their outputs, which are then converted into word features W.

**Fig 3:**
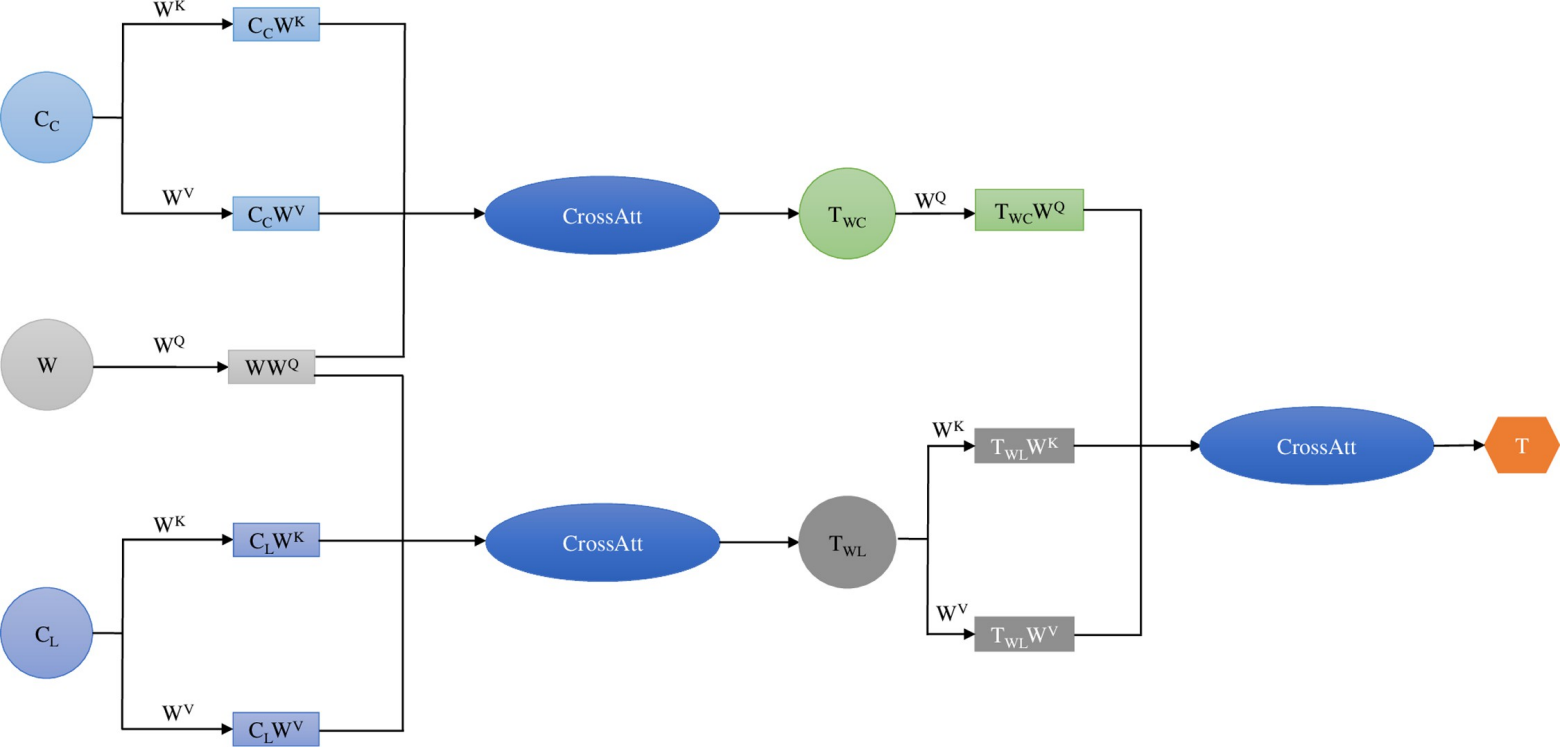
Cross attention flowchart.

Firstly, assuming that the input sentence is an X of length n, *X* = {*X*_1_, *X*_2_, …, *X*_*n*_}, where X is a sequence of words from a biomedical dataset. All words in the sentence are obtained through a pre-trained model DistilBioBERT to obtain their outputs, which are then converted into word features W. Meanwhile, the sentence is divided into a series of characters and converted to one-hot vectors and initial character embedding vectors, which are extracted for character-level features using CharCNN and CharLSTM. The character-level features *C*_*C*_ are extracted through CharCNN, while the character-level features *C*_*L*_ are extracted using CharLSTM. CharCNN uses multiple-sized convolutional kernels to simultaneously capture different-sized character features, thereby obtaining a more comprehensive and rich representation at the character level. By combining CharCNN and CharLSTM, the advantages of both can be fully utilized. CharCNN can learn character-level local features, while CharLSTM can capture character-level contextual information. Therefore, the character embedding representation simultaneously includes character-level local features and contextual information. Subsequently, two cross-attention feature fusion processes are performed. The first fusion combines the word features W with the character features *C*_*C*_ to obtain the sub-feature *T*_*WC*_. The second fusion combines the word features W with the character features *C*_*L*_ to obtain the sub-feature *T*_*WL*_. Finally, these two sub-features, *T*_*WC*_ and *T*_*WL*_, are fused again through cross-attention to obtain the final feature vector T. The specific process is shown in Fig 3. Eqs (11–13) represent the three cross-attention feature fusions.


$$\left\{\begin{array}{c} Q=WW_{q}\\ K=C_{C}W_{k}\\ V=C_{L}W_{v}\\ T_{WC}=CrossATT(Q,K,V)=softmax\left(\frac{QK^{T}}{\sqrt{d_{K}}}\right)V \end{array}\right.$$ (11)

$$T_{WL}=softmax\left(\frac{WW_{q}{\left(C_{C}W_{k}\right)}^{T}}{\sqrt{d_{C_{C}W_{k}}}}\right)C_{L}W_{v}$$ (12)

$$T=softmax\left(\frac{T^{WC}W_{q}{\left(T^{WL}W_{k}\right)}^{T}}{\sqrt{d_{T^{WL}W_{v}}}}\right)T^{WL}W_{v}$$ (13)

Among them, *W*_*Q*_, *W*_*K*_, and *W*_*V*_ are parameter matrices, and *d*_*K*_ is the dimension size of K.

### 3.3. Information extraction layer

The feature vector *T* = {*t*_1_, *t*_2_, …, *t*_*n*_}, extracted by the feature extraction layer, is input into the BiLSTM. BiLSTM is composed of a forward LSTM and a backward LSTM, which can capture the contextual information and dependencies of the input sequence. In the BiLSTM layer, each feature vector passes through a two-layer BiLSTM to capture past and future information from different directions, and then the two independent hidden states are connected to form the final context representation. The forward propagation process is as follows: Eq (14) represents the initial hidden state, Eq (15) represents the input gate, forget gate, output gate, and update unit, and Eq (16) represents the update of the cell state and hidden state.


$$ht^{\left(0\right)}=\left\{t_{1},t_{2},\ldots ,t_{n}\right\}$$ (14)

$$\left\{\begin{array}{c} i_{t}=\sigma \left(W_{i}\left[h_{t-1},x_{t}\right]+b_{i}\right)\\ f_{t}=\sigma \left(W_{f}\left[h_{t-1},x_{t}\right]+b_{f}\right)\\ o_{t}=\sigma \left(W_{o}\left[h_{t-1},x_{t}\right]+b_{o}\right)\\ {\tilde{C}}_{t}=tanh\left(W_{C}\left[h_{t-1},x_{t}\right]+b_{C}\right) \end{array}\right.$$ (15)

$$\left\{\begin{array}{c} C_{t}=f_{t}*C_{t-1}+i_{t}*{\tilde{C}}_{t}\\ h_{t}=o_{t}*tanh\left(C_{t}\right) \end{array}\right.$$ (16)

Among them, *σ* represents the activation function between basic elements, and *i*_*t*_, *f*_*t*_, *o*_*t*_, ${\tilde{C}}_{t}$, and *C*_*t*_ represent the input gate, forget gate, output gate, temporary cell state, and cell state, respectively. *W*_*i*_, *W*_*f*_, *W*_*o*_, and *W*_*C*_ are the weight coefficients for the input gate, forget gate, output gate, and temporary cell state, respectively, while *b*_*i*_, *b*_*f*_, *b*_*o*_, and *b*_*c*_ are the bias values for the input gate, forget gate, output gate, and temporary cell state, respectively.

The backward propagation process is the same as the forward propagation process but in the opposite direction. At step i, the hidden states are connected to form the final context representation, as shown in Eq (17). We can obtain a bidirectional output sequence *H* = {*h*_0_, *h*_1_, …, *h*_*n*-1_}.


$$\left\{\begin{array}{c} \vec{h_{l}}=\vec{LSTM}\left(x_{i},\;\vec{h_{l-1}}\right)\\ \overset{\leftarrow }{h_{l}}=\overset{\leftarrow }{LSTM}\left(x_{i},\overset{\leftarrow }{\;h_{l-1}}\right)\\ h_{i}=\vec{h_{l}}⊕\overset{\leftarrow }{h_{l}} \end{array}\right.$$ (17)

Where $\vec{h_{t}}\;\text{and}\;\overset{\leftarrow }{h_{t}}$ represent the hidden state information of the preceding and subsequent context at time t, respectively. *x*_*t*_ represents the input word at time t, and ⊕ denotes the concatenation operation of LSTM information vectors. *h*_*t*_ represents the hidden state output of BiLSTM at time *t*.

The bidirectional output sequence H is then used as input, and multiple attention heads are employed to capture different focuses. By introducing multiple attention heads, the model can simultaneously pay attention to different semantic information, extract feature information that plays a key role in entity recognition, and weight the output feature vectors of the upper layer, enhancing the representation ability of the input information. This can also address the challenge of BiLSTM in capturing long-distance dependencies and improve model performance. Multi-head attention mainly performs multiple different linear transformations on the query, key, and value, allowing the model to learn relevant information in different subspaces, thereby greatly improving the model’s fitting ability. Eqs (18–20) represent the calculation process of multi-head attention.


$$\left\{\begin{array}{c} Q=HW_{q}\\ K=HW_{k}\\ V=HW_{v}\\ \;Attention\;(Q,K,V)=softmax\left(\frac{QK^{T}}{\sqrt{d_{K}}}\right)V \end{array}\right.$$ (18)

$$head_{j}=Attention\;\left(Q_{j},K_{j},V_{i}\right)$$ (19)

$$MultiHead\;(Q,K,V)=Concat\;\left(head_{1},\ldots ,\;head_{m}\right)W$$ (20)

Among them, *head*_*j*_ represents the attention calculation result of the *j*^*th*^ attention head, Concat represents the concatenation of multiple heads, and W represents the weight matrix.

### 3.4. Output layer and training

This model employs an output layer to obtain the final recognition result, which comprises a first linear layer, a SiLU activation function, a dropout layer, and a second linear layer. The first linear layer is used to reduce the dimension of the feature vectors output by the multi-head attention mechanism. The SiLU activation function performs a nonlinear transformation on the output of the first linear layer. The dropout layer is utilized to prevent overfitting of the model. Finally, the second linear layer maps the nonlinearly transformed features to the category space, completing the entity recognition process.

In many natural language processing tasks, such as named entity recognition and relation extraction, there exists a serious issue of data imbalance. This imbalance mainly manifests in two aspects. Firstly, there is a class imbalance: different entity categories may have significant differences in the number of instances in the training set. This leads to the model being biased towards categories with more samples during training, neglecting the recognition of categories with fewer samples. Secondly, there is a sample imbalance: even within the same entity category, there may be an imbalance among different samples. This can result in the model overfitting to frequently occurring entities and performing poorly on rare entities. This means that the model may be more likely to apply common categories to all inputs during prediction, ignoring or misclassifying entities from rare categories. Additionally, sample imbalance can also limit the generalization ability of the model. When the model overfits to samples from common categories, it may not be able to effectively handle unseen or newly emerging entity categories, especially in practical applications where the emergence of new entities is inevitable.

To address the issue of data imbalance, the model uses both the cross-entropy loss function *L*_*CE*_, as shown in Eq (21), and the Focal Loss loss function *L*_*FL*_, as shown in Eq (22), for joint training. The final loss function Loss is shown in Eq (23).


$$L_{CE}=-\frac{1}{N}\sum_{i}\sum_{j\in \{0,1\}}y_{ij}logp_{ij}$$ (21)

$$L_{FL}={\left(1-p\right)}^{\gamma }logp$$ (22)

$$Loss=L_{CE}+L_{FL}$$ (23)

Among them, γ is the adjustable factor, which was taken as 2 in this experiment.

## 4. Experiments and analysis of results

### 4.1. Biomedical datasets

We conducted our research on five datasets in the field of biomedicine: NCBI-Disease, BC5CDR-Disease, BC5CDR-Chem, BC2GM, and JNLPBA. Table 1 shows the datasets, entity types, and their corresponding quantities used in this experiment.

1. NCBI-Disease [25] is provided by the National Center for Biotechnology Information (NCBI) in the United States and is used for identifying and annotating disease-related entities in biomedical literature.
2. BC5CDR [26] is provided by the BioCreative organization to promote the automatic extraction of relationships between chemical substances and diseases. For the BioNER task, it can also be divided into two categories: BC5CDR-Disease and BC5CDR-Chem.
3. BC2GM [27] is the dataset for the BioCreative II gene mention recognition task, which can be used to train and evaluate gene mention recognition models for biological literature processing.
4. JNLPBA [28] is derived from the GENIA 3.02 corpus and created through a controlled search on MEDLINE.

**Table 1:** Detailed representation of the dataset.

Dataset	Entity Type	Entity count
NCBI-Disease	Disease	disease: 5118
BC5CDR-Disease	Disease	disease: 4098
BC5CDR-Chem	Chemical	chemical: 5185
BC2GM	Gene	gene: 15047
JNLPBA	Gene/Protein	dna: 8392
		protein: 27032
		cell_type: 6177
		protein: 27032
		cell_type: 6177
		cell_line: 3380
		rna: 837

### 4.2. Experimental parameters

The experimental parameters for this experiment are shown in Table 2. The experimental environment is: operating system: Ubuntu 20.04, deep learning framework: PyTorch 2.0.0, development environment: Python 3.8, GPU: RTX 4090 (24GB), CPU: 15 vCPU Intel(R) Xeon(R) Platinum 8336C CPU @ 2.30GHz, memory: 80G.

**Table 2:** Experimental parameters.

Parameter name	parameter value
lr	5e-5
optimizer	Adam
epoch	15
batch size	8
convolution kernel size	4,8

### 4.3. Experimental results and comparison

This section compares our model with recent models on five public datasets, as shown in Table 3. Among them: (1) MTL-LS [29] proposed an effective hierarchical transfer learning method that combines multi-task learning and single-task learning to achieve multi-level information fusion between underlying entity features and upper-level data features. (2) PubMedBERT [30] proposed a method of specific domain pre-training from scratch and task-specific fine-tuning. This method pre-trains on a large-scale biomedical literature to generate domain-specific language representations, thereby improving the performance of the BioNER task. (3) HunFlair [31] proposed a NER tagger HunFlair that covers multiple entity types. It utilized a pre-trained language model Flair and combines it with data from the biomedical domain for named entity recognition. (4) BioDistilBERT [24] proposed a model based on BioBERT using multiple compression strategies, as many existing pre-trained models are resource-intensive and have high compute requirements. (5) BioBERT-CFA [32] proposed a new fully shared multi-task learning model based on pre-trained BioBERT, which integrates syntactic information into the BioBERT encoder using composite feature attention to improve performance. (6) PAMDFGA [33] proposed a novel and effective prefix and attention map discrimination fusion guidance attention mechanism that enhances BioBERT by changing the attention distribution.

**Table 3:** Comparison of experimental results with baseline model.

	Datasets				
Model Name	NCBI-Disease	BC5CDR-Disease	BC5CDR-Chem	JNLPBA	BC2GM
MTL-LS [29]	89.25	87.28	92.83	/	82.92
PubMedBERT [30]	87.82	85.62	93.33	80.06	84.52
HunFlair [31]	88.65	/	/	77.60	/
DistilBioBERT [24]	87.61	85.61	94.48	79.10	86.97
BioBERT-CFA [32]	89.90	87.46	93.56	78.38	85.36
PAMDFGA [33]	90.55	87.53	94.16	/	85.45
our model	**90.76**	**89.79**	**94.98**	**80.27**	**88.84**

Through experiments, we found that our model achieved the best results in terms of F1 score on five public datasets. Our model outperformed the previous record on NCBI-Disease by 0.21%, reaching a score of 90.76%, on BC5CDR-Disease by 2.26%, reaching a score of 89.79%, on BC5CDR-Chem by 0.5%, reaching a score of 94.98%, on BC2GM by 1.87%, reaching a score of 88.84%, and on JNLPBA by 0.21%, reaching a score of 80.27%. Compared to DistilBioBERT and PubMedBERT, our model incorporated CharCNN and CharLSTM to jointly extract features, enhancing its ability to extract embedded features. Compared to MTL-LS, which uses XLNet instead of BERT as the encoder and CRF as the decoder, our model uses a dual encoder DistilBioBERT and an output layer with two linear layers and an activation function to output the final result, improving its ability to model contextual information and enhancing its robustness to noise, thereby improving model performance. Compared to BioBERT-CFA, which uses composite attention to merge syntactic features into BioBERT, and PAMDFGA, which enhances BioBERT by fusing prefix and attention map discrimination guidance attention mechanisms, our model uses cross-attention to merge character features into DistilBioBERT, enhancing feature extraction performance, and uses multi-head attention for further enhancement of information extraction performance. Compared to HunFlair, which uses a character-level language model, our model uses a word-level language model while integrating character features, providing richer and more accurate representations that capture more fine-grained features and semantic information within words, improving the recognition ability of the BioNER task.

To further analyze our model, we conducted more detailed experiments on the BC5CDR-Disease dataset, and the results are shown in Table 4. Method 1 uses CharCNN and DistilBioBERT for cross-attention fusion, method 2 uses CharLSTM and DistilBioBERT for cross-attention fusion, method 3 uses CharCNN and CharLSTM for cross-attention fusion, method 4 uses only one DistilBioBERT without cross-attention fusion, and method 5 uses two DistilBioBERTs for cross-attention fusion.

**Table 4:** Cross-attention results from each module.

	P	R	F1
method 1	88.55	90.62	89.57
method 2	88.52	90.28	89.39
method 3	88.63	90.23	89.42
method 4	88.57	90.02	89.29
method 5	88.21	**90.65**	89.41
our model	**89.38**	90.20	**89.79**

Through experiments, it was found that compared to method 2, which uses CharLSTM for character feature extraction, method 1, which uses CharCNN, has higher precision, recall, and F1 values. This further indicates that CharCNN performs better when used alone as a character feature extraction model. The possible reason is that in CharCNN, the convolutional kernels share parameters across different positions in the input sequence, which helps reduce the number of model parameters and enhances the generalization ability of features. Meanwhile, compared with CharLSTM, the convolutional operations in CharCNN can be accelerated through parallelization, making CharCNN more efficient in processing large-scale text data. Method 3, compared to our model, has lower precision, recall, and F1 values, indicating that separately extracting character features using word-char fusion extraction performs better and further demonstrating the effectiveness of DistilBioBERT as a word-level feature extraction model that can capture richer and more accurate word-level representations. The possible reason is that the fusion of characters and words can simultaneously utilize the information from both, reducing the occurrence of ambiguity and thus representing the text more accurately. In some cases, individual characters or words may have ambiguous meanings, making it difficult to accurately express their intended sense. The results of methods 4 and 5 show that using a dual encoder can better model contextual information, reduce ambiguity, enhance robustness to noise, and improve performance. The possible reason is that the dual encoder provides precise location information, which helps the model accurately identify entities in the text. Furthermore, the dual encoder can enhance the modeling capability for entity position and direction information by comparing the outputs of the two encoders, further improving the performance of entity recognition.

### 4.4. Ablation experiment

We conducted ablation experiments using different fusion methods, different numbers of CharCNN convolutional kernels, multi-sized CharCNN convolutional kernels, and different numbers of multi-head attention.

#### 4.4.1. Impact of different fusion methods

This experiment mainly focuses on the fusion method of the feature extraction layer, namely, the exploration of two methods: cross-attention fusion and direct concatenation. Method 1 uses direct concatenation for all three feature fusion operations in the feature extraction layer. Method 2 proposes the use of cross-attention fusion for all three operations. Method 3 fuses W and *C*_*L*_ using cross-attention fusion, while the other two operations use direct concatenation. Method 4 fuses W and $C_{C}$ using cross-attention fusion, while the other two operations use direct concatenation. Method 5 uses direct concatenation for W, *C*_*C*_, and *C*_*L*_, and then performs cross-attention fusion. Method 6 performs cross-attention fusion for W, *C*_*C*_, and *C*_*L*_, and then uses direct concatenation.

The experimental results are shown in Table 5. When using the fusion method of method 4, the Precision is the highest. When using the fusion method of method 1, the Recall is the highest. However, when using the fusion method proposed by our model (method 2), the F1 score reaches the highest level. This indicates that our proposed three-cross-attention fusion method is beneficial for improving the recognition effectiveness of named entity recognition. The possible reason is as follows: Firstly, the cross-attention mechanism can dynamically adjust the weights between different word and character features, thereby more effectively integrating global semantic information and directional information. Secondly, the cross-attention mechanism can handle input sequences of varying lengths. Although the direct concatenation of features is simple and intuitive, it may not effectively deal with the interactions and dependencies between different features. Moreover, it may encounter difficulties when processing variable-length sequences.

**Table 5:** Experimental results of different fusion methods.

Different fusion methods	P	R	F1
method 1	88.15	**90.54**	89.33
method2	89.38	90.20	**89.79**
method3	88.48	90.41	89.43
method4	**89.54**	89.49	89.51
method5	88.59	89.97	89.27
method6	88.85	90.20	89.52

#### 4.4.2. The effect of different number of CharCNN convolutional kernels

To study the effect of different numbers of CNN convolutional kernels, we conducted a series of experiments on the BC5CDR-Disease dataset, as shown in Table 6. We found that when the number of convolutional kernels is 20, the Precision is the highest, when the number of convolutional kernels is 90, the Recall is the highest. However, when the number of convolutional kernels is 40, the F1 score is the highest. Therefore, we concluded that when the number of convolutional kernels is 40, named entity recognition performs best. Too few or too many convolutional kernels will affect its recognition performance. The reason may be that adding more convolutional kernels can capture a wider variety of features, which might describe the input data from different perspectives or levels. At the same time, multiple convolutional kernels are also more resilient to noise and variations in the input data. This enables the model to enhance its feature extraction capabilities in practical applications and handle various complex named entity recognition scenarios. However, an excessive number of convolutional kernels can lead to overfitting of the model to the training data, thereby reducing its performance on test data. Additionally, having too many convolutional kernels can also result in excessively long embedding times for character-based inputs, leading to poor performance.

**Table 6:** Experimental results with different number of charcnn convolutional verifications.

CharCNN number of convolutional kernels	P	R	F1
10	88.54	90.03	89.28
20	**89.06**	90.48	89.76
30	88.57	90.26	89.41
40	89.38	90.20	**89.79**
50	88.64	90.05	89.34
60	88.72	90.32	89.51
70	88.58	90.26	89.41
80	88.76	90.29	89.52
90	88.49	**90.60**	89.53

#### 4.4.3. Effects of multi-sized CharCNN convolution kernels

This experiment uses multi-sized convolutional kernels for character feature extraction to capture character combinations of different lengths and better understand the context features in the text, thereby improving embedding performance. In this experiment, we conducted a series of experiments on the BC5CDR-Disease dataset using one, two, and three different sizes of convolutional kernels. The final experimental results are shown in Table 7. When using one size (4), the Recall is the highest, while using two sizes (4.8) results in the highest Precision and F1 score. The experimental results demonstrate that using two sizes (4,8) results in the best performance for our model, which proves the advantage of using multi-sized convolutional kernels for character feature extraction. This may be because using multi-scale convolutional kernels allows the extraction of features from different receptive fields, thereby capturing features of different levels in the input data. This helps to improve the recognition performance of the model.

**Table 7:** Experimental results on the impact of multi-sized CharCNN convolution kernels.

	P	R	F1
4	88.55	**90.84**	89.68
6	89.03	90.44	89.73
8	88.50	90.67	89.57
4,6	88.54	90.82	89.67
4,8	**89.38**	90.20	**89.79**
6,8	89.13	90.17	89.65
4,6,8	88.22	90.76	89.47

#### 4.4.4. Impact of different amounts of multi-heads attention

Our model uses multi-head attention after BiLSTM to increase the extraction ability of feature information and solve the problem of BiLSTM in capturing long-distance dependencies. In this experiment, we studied the number of heads in multi-head attention, and the experimental results are shown in Table 8. When the number of heads is 1, the Precision is the highest, and when the number of heads is 8, the Recall is the highest. When the number of heads is 4, the F1 score is the highest. Based on a comprehensive analysis, when the number of heads is 4, the extraction ability of feature information is the strongest, and the recognition effect of NER is the highest.

**Table 8:** Experimental results on the effects of different numbers of heads.

	P	R	F1
0	89.22	90.15	89.68
1	**89.40**	89.80	89.60
2	89.23	90.04	89.63
4	89.38	90.20	**89.79**
8	88.55	**90.95**	89.73

In this regard, we believe that the increase in the number of multi-head attention heads can enable the model to simultaneously focus on different parts of the input sequence by processing multiple attention weights in parallel. This approach allows the model to encode and represent input data from more dimensions and angles, thus helping the model capture richer contextual information and improve recognition accuracy. However, when the number of heads becomes excessive, the number of parameters that the model needs to optimize will increase significantly, leading to overfitting or difficulties in training. Additionally, too many heads may result in information redundancy or interference, which can decrease the model’s performance. Therefore, in practical applications, it is necessary to select an appropriate number of heads based on specific task requirements, dataset characteristics, and computational resources. Through experiments and validation, we can find the optimal head configuration to strike a balance between performance and computational efficiency.

## 5. Conclusion and future work

In this paper, a Bi-BWC-LM model based on multi-cross attention fusion is designed. It utilizes triple cross attention fusion to obtain feature vectors, extracts information through BiLSTM and multi-head attention, and finally outputs the final results through the output layer. At the same time, a combined loss function is used to train the model. Experimental results on five datasets, including NCBI-Disease, BC5CDR-Disease, BC5CDR-Disease, BC2GM, and BC2GM, as well as ablation experiments, demonstrate that the proposed model is highly effective in improving the recognition performance of biomedical named entities.

However, according to the experiments conducted in [24], it is evident that DistilBioBERT still lags behind BioBERT by approximately 1% in terms of the F1 score. In the future, we will delve deeper into exploring ways to further reduce model parameters and enhance inference speed while leveraging BioBERT as our foundation.
